# Equipping medical hand-washing stations with cold drinking-quality water – a contribution to sustainability

**DOI:** 10.3205/dgkh000676

**Published:** 2026-09-10

**Authors:** Simone Scheithauer, Franziska Lexow, Britta Hepner, Martin Exner, Heike von Baum, Marc Thanheiser, Axel Kramer

**Affiliations:** 1 Department of Infection Control and Infectious Diseases, University Medical Center Göttingen (UMG), Georg-August University Göttingen, Göttingen, Germany; 2 Member of the Commission for Infection Prevention and Hygiene in Healthcare and Nursing (KRINKO) at the Robert Koch Institute (RKI), Berlin, Germany; 3 Robert Koch Institute (RKI), Department for Infectious Diseases, Unit 14 Hospital Hygiene, Infection Prevention and Control, Berlin, Germany; 4 Institute of Medical Microbiology and Hygiene, University Clinic Ulm, Ulm, Germany; 5 Institute of Hygiene and Environmental Medicine, University Medicine Greifswald, Greifswald, Germany

**Keywords:** cold-water handwashing, carbon footprint, hygiene/infection prevention and control benefits

## Abstract

Equipping medical handwashing stations with cold water only as a standard is recommended for the following reasons: contribution to sustainability through a reduced carbon footprint, reduced risk of *Legionella* spp. proliferation in the drinking water system, elimination of the need to test hot water for *Legionella* spp., prevention of accidental scalding, reduced skin irritation, and cost savings – all while achieving the same reduction of transient skin flora as with warm water.

## Abstract (de)

Aus folgenden Gründen wird die Standardaussattung medizinischer Handwaschplätze mit Reihung nur mit kaltem Wasser empfohlen: Beitrag zur Nachhaltigkeit durch Reduzierung des CO_2_-Fußabdrucks, Reduzierung des Risikos der Vermehrung von *Legionella* spp. in der Trinkwasserinstallation, Entfallen der Untersuchung von Warmwasser auf Legionellen, Vermeidung akzidenteller Verbrühung bei gleicher Abreicherung der transienten Hautflora wie durch Warmwasser, Reduzierung der Hautirritation und Kostenreduktion.

## Introduction

Previous German recommendations stated that medical handwashing stations for employees must be equipped with inlets for both hot an cold water [1], [2]. Recent updates recommend “water of drinking-water quality” without the need to provide hot water [3].

This is in line with the general assessment by the World Health Organization (WHO) and the US Centers for Disease Control and Prevention (CDC), that the effects of water temperature on effective depletion of pathogens from the hands are negligible [4], recently conformed in an in vitro study [5].

## Benefit and risk assessment

The elimination of the obligation to provide hot water offers opportunities for proportionate solutions for a wide variety of facilities and functions for reasons of resource and environmental conservation as well as cost savings. Benefits are summarized in the infographic. Additionally, in times of climate crisis, hybrid attacks, and war, a power outage must always be expected. In such situations, you can only wash with cold water. This needs to be taken into account more when weighing risks. Climate change has been identified as the greatest global threat to human health in the 21^st^ century and has been described by the Intergovernmental Panel on Climate Change (IPCC) as an existential threat to humanity [6]. Globally, the healthcare sector itself contributes significantly to the climate crisis, being responsible for 4.4% of total climate-damaging emissions [7]. The WHO sees significant potential for the healthcare sector to actively contribute to climate protection through more sustainable structures and processes [8]. The carbon dioxide balance (CO_2_ footprint) can be reduced safely from an infection prevention point of view by promoting the use of cold water. If, for example, 100 handwashing activities per day were carried out in a hospital over one year using only cold water, between 1.5 and 2.1 tonnes of CO_2_ could be saved, depending on the proportion of fossil fuels (e.g. gas, oil, electricity). In Germany, one litre of hot water costs about 2.5 to 4 times as much as a litre of cold water, depending on the type of energy source. Thus, this intervention is part of the national infection prevention and control (IPC) recommendations, making IPC more sustainable [9].

Not providing hot water can also reduce the risk of accidental scalding. With regard to water temperature, the comfort aspect of a pleasant water temperature probably increasing adherence to hand washing needs to be taken into account. However, the most important single measure of hand hygiene in medical and nursing care is hand antisepsis using alcohol-based hand rubs. Handwashing is primarily used for mechanical cleaning of the hands such as at the start of the day of surgery and for certain indications, e.g. the care of patients with *Clostridioides (C.) difficile* infections (CDI). The effectiveness of handwashing on the transient flora correlates with the duration of handwashing, the technique, the quality of the water used and the handwashing preparations, but not with temperature [5], [10], [11]. The experimental comparison of rinsing the hands with cold vs. warm water regarding the reduction of contamination with *Escherichia (E.) coli* K 12 showed no difference using EN 1499 as method [11]. The authors conclude that avoiding hot water is a safe and resource-saving method [5]. Even more, skin irritation by washing hands and especially using hot water [12], [13] can have a negative impact on the implementation of hygienic hand antisepsis and thus increase the likelihood of pathogen transmission and infection among the employees themselves, respectively.

Education about the benefits of resource conservation can help ensure acceptance. Furthermore, the removal of the obligation to provide hot water is not to be equated with a ban. However, using only cold water could – especially with regard to climate change – reduce the risk of the presence, multiplication and cross-contamination of *Legionella* spp. and thus the risk of infection. The growth of virulent Legionella is favoured in the temperature range of 25°C–50°C, which is why the hot water temperature should be at least 55°C and the cold water temperature below 25°C according to technical regulations. Ensuring a hot water temperature of 55°C at least requires a huge amount of energy. There is no alternative to temperature for safely controlling Legionella in hot water, as chemical disinfection methods in hot water are not sufficiently reliable, and there are always reports of outbreaks despite adequately high disinfectant concentrations. Therefore, the strategy of focusing hot water supply only on areas where it is truly needed from a hygienic-medical perspective, is sensible and represents a significant contribution to energy efficiency.

When considering a change to cold-water supply only in existing buildings, caution is required. If existing hot water inlets are not or seldom used, a regular flushing of the hot water pipe must be included in the facility's internal water safety plan [14]. Alternatively, a professional dismantling of the hot water pipes can be considered. The intervention perfectly fits to new buildings helping to decrease risks, the carbon footprint and costs by one single intervention.

## Notes

We want to acknowledge all other KRINKO members and the KRINKO office members at the RKI as well as the secretary office of the Department of Infection Control and Infectious Diseases, UMG.

### Authors’ ORCIDs

- Scheithauer S:
https://orcid.org/0000-0003-0773-4739
- Lexow F: https://orcid.org/0000-0003-3463-2842
- Hepner B: https://orcid.org/0009-0002-5660-157X
- Exner M: https://orcid.org/0000-0002-6383-7866
- Thanheiser M:
https://orcid.org/0009-0005-4139-6544
- Kramer A: https://orcid.org/0000-0003-4193-2149

### Funding

None. 

### Competing interests

The authors declare that they have no competing interests.

### AI usage statement 

No artificial intelligence (AI) tools were used. All aspects of the work were carried out solely by the authors.
